# Seasonal, Diurnal and Vertical Variation of Chlorophyll Fluorescence on *Phyllostachys humilis* in Ireland

**DOI:** 10.1371/journal.pone.0072145

**Published:** 2013-08-15

**Authors:** Davina Van Goethem, Sebastiaan De Smedt, Roland Valcke, Geert Potters, Roeland Samson

**Affiliations:** 1 Department of Bio-science Engineering, University of Antwerp, Antwerp, Belgium; 2 Laboratory for Molecular & Physical Plant Physiology, Hasselt University, Hasselt, Belgium; 3 Antwerp Maritime Academy, Antwerp, Belgium; University of California - Davis, United States of America

## Abstract

In recent years, temperate bamboo species have been introduced in Europe not only as an ornamental plant, but also as a new biomass crop. To measure adaptation stress of bamboo to the climate of Western Europe, chlorophyll fluorescence was measured on a diurnal and seasonal basis in Ballyboughal, Co. Dublin, Ireland. Measurements were attained on the leaves of each node of *Phyllostachys humilis*. The most frequently used parameter in chlorophyll fluorescence is the photosynthetic efficiency (Fv/Fm). A seasonal dip - as well as a larger variation - of Fv/Fm in spring compared to the rest of the year was observed. Over the year, the upper leaves of the plant perform better than the bottom leaves. These findings were linked to environmental factors such as light intensity, air temperature and precipitation, as increased light intensities, decreasing air temperatures and their interactions, also with precipitation levels have an effect on the photosynthetic efficiency (Fv/Fm) in these plants.

## Introduction

Bamboo is a widespread woody grass with over 1200 species and 90 genera [1], covering large areas north and south of the Equator. However, like most woody plants, bamboos have failed to colonize the harshest environments, such as the arid desert or Arctic tundra. Due to the last ice age, Europe and most of North America were thus excluded from the potential areas where bamboos could establish. However, temperate bamboos are growing in these two continents and, if climatic history had been different, these plants may well have been native in Europe and North America [2].

Although some of them are extremely fast-growing, bamboo species have largely been neglected in the search for renewable energy sources [3]–[5]. However, despite the fact that they are not native to this continent, in recent years, there is a growing interest for these exotic plants in Europe [6]. On top of their potential as biomass producer, bamboo can also be used for renewable bio-based materials such as wood, composites, vessels and chemicals [3], [7]–[9] and for the removal of pollutants - such as heavy metals - out of the soil [5].

The bamboo genera most suitable in Europe are temperate bamboos such as *Sasa, Fargesi*a and *Phyllostachys*. Among these, *Phyllostachys* species are known to have a very high annual biomass production [3], and they can survive temperatures as low as −20°C [10]. These temperate bamboos can be cultivated in Europe, however, it is still unknown how these plants respond and develop in this foreign environment. To assess the possible damages of different (environmental) stress signals, fluorescence spectroscopy can be used [11].

The shape of the fluorescence transient of any sample is determined by the physiological state of the sample at that moment and the physical and chemical environmental conditions around the sample [12], such as light intensity [13]–[15], temperature [16]–[18], drought [19] or chemical influences [20]. A light-induced decline of photosynthetic activity is broadly termed as photoinhibition [21]. It is a process taking place when the photosynthetic apparatus receives excess excitation energy and is hence called the excitation pressure hypothesis [22]–[24]. The site of inhibition of photosynthesis is photosystem II [25] and photoinhibition characteristically causes a loss of variable chlorophyll fluorescence [26]. One of the most frequently used parameters in chlorophyll fluorescence is the ratio between variable and maximum fluorescence (Fv/Fm). Across a wide range of higher plants species, this parameter has an optimal value of 0.83. When exposed to abiotic and biotic stresses, Fv/Fm in plants will decrease [27]. This parameter is therefore frequently used as a stress detector under environmental stress conditions, such as light [28], temperature [24] and drought [29].

Seasonal dynamics in chlorophyll fluorescence have been reported for different species. Depressions in photochemical efficiency (Fv/Fm) were observed in winter in Mediterranean shrub species [30], [31], during cold winter days in conifer species [32]–[35] or in spring and early summer in Mediterranean grassland species [36] when ambient temperatures were increasing rapidly and precipitation was low. Aside from seasonal changes, chlorophyll fluorescence can also vary between sun-exposed and shaded leaves [37]–[41]. Also midday depression of photochemical efficiency is common in higher plant species like in Mediterranean grassland species [42] and in other bamboo species [43].

The seasonal dynamics in chlorophyll fluorescence in other plants raise the hypothesis that the photochemical efficiency in *Phyllostachys humilis* in Ireland would be lower in winter or in spring and early summer compared to the rest of the year. Also a midday depression can be expected, as this is observed in other higher plant species. These variations in photochemical efficiency will probably be correlated with environmental factors such as light intensity, air temperature and precipitation. Since lower leaves are less exposed to microclimatic variations in the environment compared to upper leaves, we also hypothesize that the lower the leaf in the canopy, the higher the photochemical efficiency will be.

Therefore, the objective of this study was to investigate seasonal, diurnal and vertical variation of Fv/Fm, in relation to light intensity, air temperature and precipitation on the bamboo *Phyllostachys humilis* in Ballyboughal, Ireland.

## Materials and Methods

### 1. Plant Material

In April 2005, a 5 m×80 m strip of *Phyllostachys humilis* was planted at a distance of 2 m within rows and 1.8 m between rows on a clay soil in Ballyboughal, co, Dublin, Ireland (N 53°31′41″ E 6°15′31″). The land accessed was privately owned and the owner of the land gave permission to conduct the study on this site. No protected or endangered species were sampled.

We selected *Phyllostachys humilis* since it is a temperate bamboo species, well suited for a temperate climate such as this of Ireland, and for his high biomass production [44]. Plants were produced via *in vitro* techniques and delivered by Oprins Plant NV (Rijkevorsel, Belgium) and were planted after hardening during one growing season in a 2 litre pot. At the moment of planting, the average height was about 50 cm. No fertilizer was added. During the first two years after planting, the plants were regularly watered during summer and Simazine© was used to suppress upcoming weeds. Additional water or spraying with herbicides was unnecessary from the third year onwards.

### 2. Data Collection

Field data were collected from different culms each season, 3 culms during summer (day of year ( = DOY) 210–216) and 3 culms during autumn (DOY 286–297) of 2009; 12 culms during spring (DOY 119–135), 8 culms during summer (DOY 197–202), 14 culms during autumn (DOY 274–288) of 2010, 8 and 18 culms during two periods in the winter of 2011 (DOY 13–25 and DOY 53–56 resp.); and 15 culms during spring (DOY 96–105) and 20 culms during autumn (DOY 284–302) of 2011. On each culm, at least three leaves on each node were measured in random order. The nodes were numbered from top (NN = 1) to bottom (NN = 13), as we were interested in the vertical effect of depth in the canopy. The maximum node number sampled was 13. Measurements were taken with the Handy PEA (Hansatech instruments Ltd., England, Norfolk) after dark adaptation with leaf clips for 30 minutes. Biolyzer HP 3.0 (Fluoromatics Software) was used to calculate Fv/Fm as an indication of leaf photosynthetic performance.

In spring, summer and autumn of 2012, LAI-2000 measurements were performed under over-cast conditions as advised by [45] to reduce the effect of scattered blue light in the canopy. Above and below canopy measurements were performed at five randomly chosen locations in the study plot.

Besides with the LAI-2000 light intensity in the canopy was also measured with the TRAC (Tracing Radiation and Architecture in Canopies [46]). TRAC measurements were performed along 2 transects over 12 m oriented in an E-W direction (to avoid boundary effects of the edge of the field). All TRAC measurements were taken under clear sky conditions to allow uninterrupted operation. The operator always held the TRAC in balance when operating and walked with a reasonable constant speed, also advised by [45] The LAI (Leaf Area Index) was calculated as the mean of the LAI-values obtained via these two instruments, as recommended by [45] for an accurate LAI estimation.

**Figure 1:**
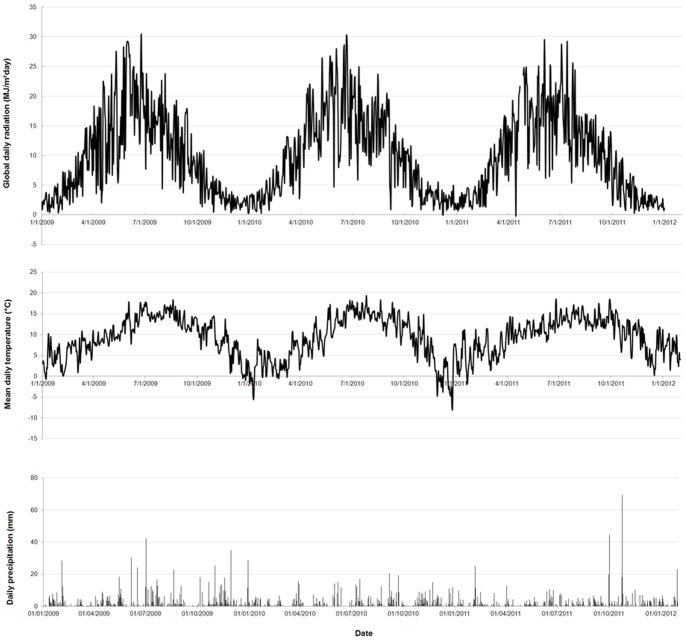
Meteorological data. Sum of the daily global radiation, mean daily air temperature and daily precipitation for 2009, 2010, 2011.

Hourly values of global radiation, air temperature and precipitation for the period from 01/01/2009 till 31/12/2011 were available from Dublin Airport, less than 12 km distant from the field (Figure 1). Meteorological conditions were comparable over the three years, with maximum global daily variation values reached in early summer (25 to 30 MJ/m^2^day), mean daily air temperatures ranging from 0 to −7°C in winter and up to 17°C in summer, and a mean monthly precipitation of about 70 mm (Fig. 1).

### 3. Statistical Analysis

#### 3.1. Seasonal, diurnal and vertical variation

In order to analyse the seasonal, diurnal and vertical variation, a statistical mixed model is used. Although harmonic functions are often used to model temporal data [47], the use of a nonlinear function is a better representative for describing Photosystem II (PSII) efficiency in the leaves of *Phyllostachys humilis* [48]. Therefore, data were analysed in the statistical program R v2.13.0 [49] using a nonlinear mixed model [50] with leaf nested in node nested in culm with the following function:
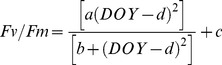
(1)with DOY = day of year, parameter a is the amplitude of the Fv/Fm values (the maximum Fv/Fm-value minus the minimum Fv/Fm-value), b is an indicator of the width of the seasonal dip (expressed in DOY^2^), c is the minimum Fv/Fm value and d is the DOY where the minimum Fv/Fm value was reached (DOY_min_). We tested the effect of TOD (time of the day) and node number (NN) and the two-way interaction of both factors on this model. The effect of TOD was tested for three moments during the day: morning, midday and afternoon; with morning measurements taken between 8 am and 10 am, midday measurements between 12 pm and 2 pm, and afternoon measurements between 4 pm and 6 pm, all expressed in local time.

Variance functions allowing for different standard deviations (for season as well as for node number) were used as described by Pinheiro & Bates (2000). They were both optimized based on the Akaike Information criterion (AIC) [51].

#### 3.2. Effect of light, air temperature and precipitation

Since the seasonal, diurnal and vertical effects are possibly correlated with environmental conditions, a linear mixed model [50] with leaf nested in node nested in culm was used to test the effect of light, air temperature and precipitation and their interactions on Fv/Fm. Since we expect the adaptation of the photosynthetic system to be rather slow [35], the mean air temperature and the sum of precipitation and global radiation were taken over the last 30 days prior to the measurement. Penetrated solar radiation at different node depths was estimated based on LAI-2000 measurements, assuming an exponential decrease in radiation with the depth in the canopy. Of each meteorological variable, the amount of variance accounted for in the population was estimated by the omega squared (Ω^2^) [52].

Variance functions allowing for different standard deviations for season were used as described by Pinheiro & Bates (2000), optimized based on the Akaike Information criterion (AIC) [51].

## Results

### 1. LAI

Mean LAI of the bamboo canopy was found to be 7.2±1.7 m^2^ m^−2^, with no significant differences between the different seasons (Table 1).

**Table 1:** Mean LAI-values (m^2^m^−2^) in spring, summer and autumn of 2011.

Season	Value	SE
Spring	7.33^a^	2.50
Summer	7.28^a^	1.52
Autumn	6.94^a^	0.96

SE = standard error. Different letters in superscript denote significant (p<0.05) differences between seasons.

### 2. Seasonal, Diurnal and Vertical Variation

**Figure 2:**
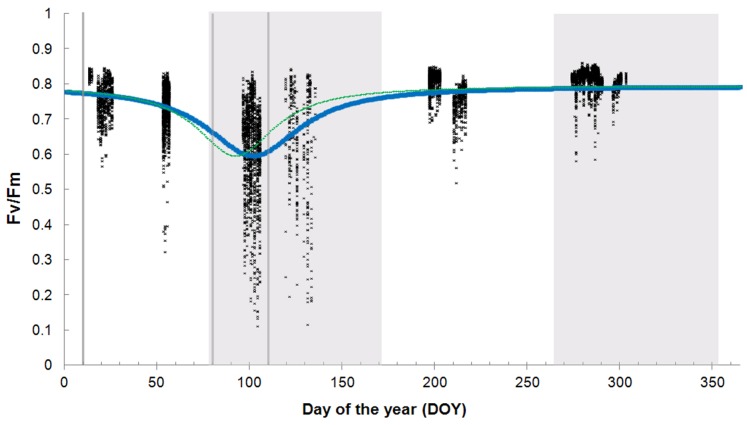
Fv/Fm (dimensionless) in function of day of year (DOY). The small crosses represent all measured values during the period 2009–2011, the small line is the fit of the nonlinear function of the 5^th^ node (NN = 5) in the morning, the thick line is the fit of the nonlinear function of the same node (NN = 5) at noon. Spring and autumn are indicated in grey, whereas winter and summer are indicated in white. The vertical bars are DOY taken for figure 3 and 4.

A depression in Fv/Fm can be clearly observed in spring, a recovery in summer and high values in autumn and winter, as fitted by the nonlinear function (Figure 2). Also the variance within each season showed large differences between seasons. The variance in Fv/Fm was small in summer, autumn and winter (var (Fv/Fm) = 0.0004, 0.0020 and 0.0026 resp.). Towards the end of the winter the variability in Fv/Fm increased (var (Fv/Fm) = 0.0041), reaching maximum values in spring (var (Fv/Fm) = 0.0187) (Figure 2). Also the variance of Fv/Fm in node number differs and is higher in the top leaves (var (Fv/Fm) = 0.0109 in the leaves of the top 2 nodes) compared to the bottom leaves (var (Fv/Fm) = 0.0104 in the leaves of the bottom 2 nodes), and this is even more explicit in summer, autumn and winter than in spring.

Node number has only an effect on the amplitude of the Fv/Fm values (parameter a in eq.1), while daytime has a significant effect on the amplitude of the Fv/Fm values, the width of the seasonal dip and the DOY_min_ (parameters a, b, and d in eq. 1, respectively) (Table 2). No significant effect was found on the minimum of Fv/Fm (parameter c in eq.1).

**Table 2:** Anova table of the seasonal diurnal and vertical effect on Fv/Fm in the nonlinear mixed model.

Parameter	Effect	numDF	denDF	F-value	p-value
A	(Intercept)	1	6851	57568.18	<0.001
	NN	1	6851	10.22	0.001
	TOD	2	6851	36.39	<0.001
B	(Intercept)	1	6851	3912.78	<0.001
	TOD	2	6851	225.52	<0.001
C	(Intercept)	1	6851	10843.96	<0.001
D	(Intercept)	1	6851	1723.03	<0.001
	TOD	2	6851	12.5	<0.001

a = amplitude of the Fv/Fm values; b = indicator of the width of the seasonal dip (expressed in DOY^2^); c = minimum Fv/Fm value;d = day of the year where the minimum Fv/Fm value was reached. NN = node number and TOD = time of the day. NumDF = numerator degrees of freedom; denDF = denominator degrees of freedom.

**Figure 3:**
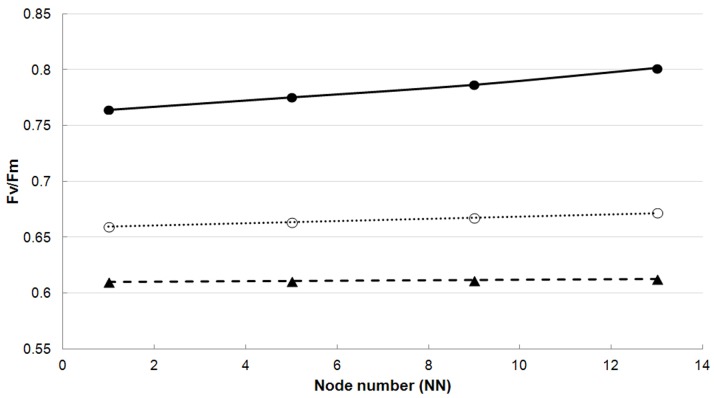
Fv/Fm (dimensionless) in function of node number (NN) at noon. Nodes are numbered from top (NN = 1) to bottom (NN = 13), Closed circles represent the predicted values for DOY 10, open circles represent the predicted values for DOY 80 and triangles represent the predicted values for DOY 110.

**Figure 4:**
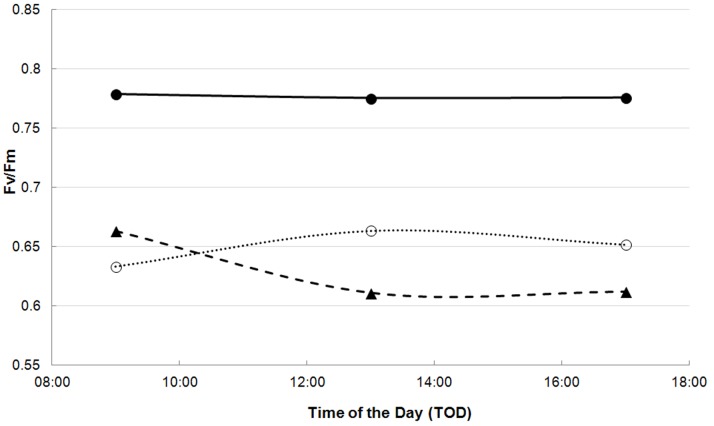
Fv/Fm (dimensionless) in function of time of the day (TOD) of the 5^th^ node. Closed circles represent the predicted values for DOY 10, open circles represent the predicted values for DOY 80 and triangles represent the fitted values for DOY 110. TOD is expressed in local time.

Fv/Fm is lower in the top leaves than in the leaves more deeply in the canopy (the effect of NN on the amplitude of Fv/Fm (parameter a in eq.1) is a positive value, and this parameter represents higher values in summer, autumn and winter) (Table 3, Figure 3). Maximum Fv/Fm values (in summer, autumn and winter) are higher in the afternoon than in morning and midday, with no statistical significant difference between morning and midday (the magnitude of the effect of TOD_pm_ on parameter a is 2–3% higher than the magnitude of the effect of TOD_am_ and TOD_noon_ on this parameter (Table 3). In spring, minimum values of Fv/Fm (parameter d of eq.1) are first observed for the morning measurements (DOY = 92) (Figure 4). For the midday and afternoon measurements, these minimum Fv/Fm values (parameter d) are only reached at DOY = 101 (Table 3). Also, the depression of the dip is less wide for the morning measurements compared to the midday and afternoon measurements (parameter b of eq.1 in table 3).

**Table 3:** Estimated values, standard error and p-value of the diurnal and vertical effect on the Fv/Fm parameter in the nonlinear mixed model.

	a		b		c		d	
	Value	SE	Value	SE	Value	SE	Value	SE
NN	3.1×10^−3^	0.72×10^−3^	NS	NS	NS	NS	NS	NS
TOD_am_	0.184^a^	0.00704	665.0^a^	71.5	0.598^a^	0.006	92.0^a^	2.50
TOD_noon_	0.182^a^	0.00712	955.0^b^	116.0	0.598^a^	0.006	101.8^b^	2.94
TOD_pm_	0.188^b^	0.0071	1184.8^b^	119.9	0.598^a^	0.006	100.6^b^	2.70

a = amplitude of the Fv/Fm values; b = indicator of the width of the seasonal dip (expressed in DOY^2^); c = minimum Fv/Fm value; d = day of the year where the minimum Fv/Fm value was reached. NN = node number and TOD = time of the day, with TOD_am_ = morning, TOD_noon_ = midday and TOD_pm_ = afternoon, SE = Standard error.

Values followed by the same letter within a column are not significantly different from each other at p<0.05. NS = not significant.

### 3. Effect of Light, Air Temperature and Precipitation

Only the effect of precipitation on the photochemical efficiency Fv/Fm was not significant (p = 0.45). All other effects (the effect of light intensity, air temperature and all two-way interaction) on the photochemical efficiency Fv/Fm were significant at p<0.001 (table 4). The negative estimated value of light means that increasing radiation intensity will result in a lower Fv/Fm-value. Air temperature has a positive effect on the chlorophyll fluorescence efficiency, as well as precipitation. The interaction between light and air temperature is positive, meaning that the negative effect of an increasing radiation on Fv/Fm is less under increasing air temperature.

**Table 4:** Estimated values, standard error (SE), p-value and omega squared (Ω^2^) of the effects of light, air temperature and precipitation and their two-way interactions on Fv/Fm.

Factors	Estimatedvalue	SE	p-value	Ω ^2^
(Intercept)	0.67	0.02		
Light	−6.5×10^−6^	0.58×10^−6^	<.0001	0.015
Air temperature	1.8×10^−2^	0.14×10^−2^	<.0001	0.156
Precipitation	4.7×10^−3^	0.21×10^−3^	0.45	0.017
Light:Air temperature	4.9×10^−7^	0.49×10^−7^	<.0001	0.078
Light:Precipitation	−2×10^−8^	0.2×10^−8^	<.0001	0.004
Air temperature:Precipitation	−3.3×10^−4^	0.16×10^−4^	<.0001	0.019

The interaction between light and precipitation is negative, meaning that the negative effect of an increasing radiation will be higher when precipitation level is increasing as well, however, the size effect of this parameter is very low (Ω^2^ = 0.004), meaning that this factor is not accounting for much of the explained variation. The interaction between air temperature and precipitation is also negative, meaning that the positive effect of high air temperature will lessen upon elevated precipitation levels.

## Discussion

### 1. General Effect of Light, Air Temperature and Precipitation

Since *Phyllostachys humilis* is a bamboo species native to the continental temperate climate of China [1], we did not expect the temperate weather condition of Ireland to be stressful for these plants. Intriguingly, we did found a significant effect of light, air temperature, precipitation and an interaction between these factors on chlorophyll fluorescence.

Air temperature has the largest effect on Fv/Fm (Table 4), with lower Fv/Fm values under decreasing air temperatures, indicating that decreasing air temperatures are stressful for the *P. humilis* plants. In literature, the negative effect of low air temperatures on Fv/Fm values has been previously reported in evergreen conifers, where reduced photochemical efficiency during winter was related to damage of the PSII reaction centers [39], [53]–[55].

Beside the effect of air temperature, we found that increasing light intensities reduces Fv/Fm in the leaves of *P. humilis*. Light stress is often referred to as an important stress factor that can induce photoinhibition [28], [56], [57]. When the amount of absorbed radiation exceeds the ability of the photosynthetic apparatus to use it, the surplus energy has to be quenched in order to avoid photo-oxidative damage to the thylakoid membranes [58]. In their natural habitat, bamboo leaves are sun-protected, since many bamboo species are typical understory tree-grasses in Japanese, Chilean and Chinese temperate and subalpine forests [59]–[64]. Although mostly *Sasa*, *Chusquea* and *Bashania* are described as understory genera [65], *P. humilis* is also found to be originated in the temperate Chinese forests [1], which might explain its sensitivity for light.

An important interaction was observed between light and air temperature, as the interaction amplifies the effect of these two factors. This means that the negative effect of increased light intensity on Fv/Fm is even stronger at decreasing air temperatures, whereas at increasing air temperatures, the negative effect of increasing light intensities will be less. Together with the main effects of light and air temperature, this is consistent with the excitation pressure hypothesis. Photoinhibition will take place when the photosynthetic apparatus receives excess excitation energy, and at low temperatures, this may occur under quite low light levels [22], [23]. Although their size effect is lower (Ω^2^ = 0.004 and 0.019, respectively) compared to the former interaction, the interactions between light and precipitation, and between air temperature and precipitation were also found to be significant. In combination with increased light intensities, and in combination with increased air temperature levels, the non-significant positive effect of precipitation on Fv/Fm will be lower, as under the climatic conditions of Ireland, light and temperature effects are more important than the precipitation effect as indicator of soil moisture deficit. However, in more precipitation limited regions, the relative importance of these effects might change.

### 2. Seasonal Variation

In the bamboo plantation, a clear seasonal depression of chlorophyll fluorescence was found in spring. This spring dip has also been found in Mediterranean grassland species [36]. The declined Fv/Fm values in spring might be explained by a combination of low air temperatures and increasing light irradiation (fig. 1), as photoinhibition can occur under these conditions [35], [38]. The increased quenching probably results from downregulation and increased zeaxanthin concentrations within the thylakoid membrane [66]. Another explanation might be that soil water availability is more important during culm development, which starts in spring [10], than in the rest of the year. As the soil water availability is linked with precipitation level, it might be the precipitation level in spring is too low, and can cause stress to the plants.

### 3. Diurnal Variation

Although midday depression has been found in other bamboo species like *Guadua angustifolia* and *Bambusa tulda* [43], no midday depression was observed in *Phyllostachys humilis*, just as in other bamboo species such as *Dendrocalamus gigantus* en *D. strictus* [43]. However, as observed by Kumar *et al.* (2002), Fv/Fm can be lower in the morning, with a recovery later during the day. In *P. humilis*, this effect was only found in early spring. In late spring, however, the reverse effect was observed (fig. 4). The lower values of Fv/Fm in the morning might be explained by the lower air temperature in the morning compared to the noon and afternoon. The reverse effect (higher values in the morning compared to the rest of the day) can be explained by the recovery from photoinhibition as nights are warmer in late spring. Other grasses that are able to tolerate cold air temperature also show rapid photosynthetic recovery when air temperatures rise in spring [67].

### 4. Vertical Variation

The negative effect of increased light intensities on Fv/Fm is also reflected in the significant effect of the depth of the leaves in the canopy. The leaves more deeply in the canopy have a higher Fv/Fm than leaves located at the top. A similar effect has been reported in boreal conifers, where a higher proportion of damaged reaction centers was found in sun-exposed than in shaded needles [40], [41], [68], [69]. Also in the evergreen shrub *Mahonia repens*, sun-exposed leaves have a lower photosynthetic efficiency during winter, whereas shade leaves did not [70]. The deeper located leaves are more protected from the sun, due to the high LAI of *Phyllostachys humilis* of 7.2±1.7 m^2^m^−2^. This LAI value is comparable with that of *P. pubescens*, where a value of 8.0 m^2^m^−2^ was measured [71]. For *P. bambusoides* [72] even observed a value of 11.6 m^2^m^−2^. Therefore, in mature stands, transmission of light decreases rapidly from the top of the bamboo canopy towards the bottom [73]. In this way, the dense canopy protects the bottom leaves against increasing radiation.

### Conclusions

We found that environmental factors as high light intensity and low air temperature can have a negative impact on Fv/Fm, representing the photochemical efficiency of the leaves of *P. humilis*, even in the temperate climate of Ireland. These effects of the environmental weather conditions explain the occurrence of a seasonal, diurnal and vertical variation in Fv/Fm of our data.

Notwithstanding the spring dip, the photochemical efficiency of the *P. humilis* leaves was generally not lower than what we would expect under optimal conditions (Fv/Fm was not much lower than 0.8). Therefore, we conclude that bamboo was not under environmental stress under these temperate conditions and thus, their potentials as a viable (biomass)crop can also be exploited in non-native European environments.

As the photochemical efficiency of the leaves is highest under shaded conditions, it might be interesting to use bamboo in agroforestry systems, by cultivating other plants (trees) above the bamboo canopy to higher the yields. However, in practice, *Phyllostachys* spp. are mostly cultivated in open, sunny situations [1]. Moreover, a higher photochemical efficiency does not necessarily mean a higher biomass production, since competition can arise respective to water use, and although a correlation with the maximum light-saturated photosynthetic rate is frequently observed [74]–[76], a low photochemical efficiency does not necessary result in a low photosynthetic activity. Therefore, gas exchange measurements should be executed to find out if high light intensities and low air temperatures and precipitation affect the overall photosynthetic activity in the same way as they influence Fv/Fm.
