# Correction: Seasonal, Diurnal and Vertical Variation of Chlorophyll Fluorescence on *Phyllostachys humilis* in Ireland

**DOI:** 10.1371/annotation/0c77f560-2ec9-4354-8ba6-5fdb8d6012ae

**Published:** 2013-11-20

**Authors:** Davina Van Goethem, Sebastiaan De Smedt, Roland Valcke, Geert Potters, Roeland Samson

The correct version of the Funding statement should read "The Special Research Fund (BOF) partly funded this research. The University Foundation of Belgium also provided funding for this research. The funders had no role in study design, data collection and analysis, decision to publish, or preparation of the manuscript." 

